# Predictive validation of qualitative fibrosis staging in patients with chronic hepatitis B on antiviral therapy

**DOI:** 10.1038/s41598-019-51638-3

**Published:** 2019-10-30

**Authors:** Hye Won Lee, Kiyong Na, Seung Up Kim, Beom Kyung Kim, Jun Yong Park, Ji Hae Nahm, Jung Il Lee, Do Young Kim, Sang Hoon Ahn, Kwang-Hyub Han, Young Nyun Park

**Affiliations:** 1 Department of Internal Medicine, Yonsei University College of Medicine, Seoul, Korea; 2 Institute of Gastroenterology, Yonsei University College of Medicine, Seoul, Korea; 3 Yonsei Liver Center, Severance Hospital, Seoul, Korea; 4 Department of Pathology, Kyung Hee University College of Medicine, Seoul, Korea; 5 Department of Pathology, Yonsei University College of Medicine, Seoul, Korea; 6 Gangnam Severance Hospital, Yonsei University College of Medicine, Seoul, Korea

**Keywords:** Gastroenterology, Liver diseases

## Abstract

The fibrosis in chronic hepatitis shows dynamic changes during antiviral therapy (AVT). We investigated whether P-I-R (progressive vs. indeterminate vs. regressive) staging is predictive of hepatocellular carcinoma (HCC) recurrence in patients with chronic hepatitis B (CHB) taking AVT who underwent resection. Patients with CHB-related HCC who underwent curative resection between 2004 and 2017 and had received ≥2 years AVT at the time of resection were eligible. Two pathologists performed P-I-R staging. In total, 104 patients with CHB-related HCC were enrolled. The mean age of the study population was 56.3 years. The mean duration of AVT at the time of resection was 62.6 months. During the follow-up period (mean, 45.5 months), 20 (19.2%) and 14 (13.5%) patients developed early and late recurrence of HCC, respectively. The cumulative incidence of late recurrence was significantly lower in patients with regressive patterns than in those with indeterminate and progressive patterns according to P-I-R staging (*P* = 0.015, log-rank test), although the cumulative incidence of overall recurrence according to P-I-R staging was similar. Hepatitis B virus DNA levels (hazard ratio [HR] = 3.200, *P* = 0.020) and the regressive P-I-R staging pattern (HR = 0.127, *P* = 0.047) independently predicted the risk of late recurrence. One-time assessment of the P-I-R staging at the time of curative resection in patients with CHB-related HCC receiving AVT independently predicted late HCC recurrence. Therefore, qualitative fibrosis assessment by P-I-R staging might be useful in predicting the outcomes of patients with CHB undergoing AVT.

## Introduction

Several staging systems, such as the Ishak, Metavir, and Batts systems, have been developed to evaluate the quantity of fibrosis and have been used to assess the degree of liver fibrosis in patients with chronic viral hepatitis^1–4^. The Metavir staging system and the Batts and Ludwig visual interpretations have obvious value due to their simplicity^5^. The Ishak staging system is beneficial because it has a greater score range, which allows more precision when classifying the degree of scarring^5^. In addition, histological subclassification of cirrhosis using the Laennec system can be more informative to assess the risk of developing liver-related events including hepatic decompensation, hepatocellular carcinoma (HCC) and liver-related death in patients with cirrhosis^6^.

However, several issues have arisen regarding these semi-quantitative staging systems for liver fibrosis that render them less applicable in this era of using active and potent antiviral therapy (AVT) for patients with chronic hepatitis B (CHB). First, because these staging systems were established based on histological changes in untreated patients, their applicability in patients with CHB receiving AVT is not well known. Second, these systems do not explain the dynamic changes in the fibrotic burden after the initiation of AVT. For example, prolonged AVT with potent antiviral agents induces fibrosis regression in patients with CHB, whereas a small proportion of patients experience progression of fibrosis despite AVT^7–10^. Third, these staging systems rarely have a role in the prediction of long-term prognosis according to the changed fibrotic burden after prolonged AVT. Some studies have demonstrated that changes in the fibrotic burden assessed by noninvasive surrogates, such as transient elastography, are significantly associated with long-term outcomes in patients with CHB^11–14^.

Recently, Sun *et al*.^15^ proposed a new staging system for the qualitative evaluation of changes in liver fibrosis based on histological results from paired biopsies performed during entecavir-based AVT, so called “Beijing classification”, enables the stratification of patients with CHB into three groups with different likelihoods of change in fibrotic burden: fibrosis progression, fibrosis indeterminate, and fibrosis regression (P-I-R staging)^15^. Although P-I-R staging may provide additional information regarding the potential for regression or progression of fibrosis^16^, no external validation study has been performed. In addition, because the P-I-R staging system was established based on the results of paired biopsies, which are not feasibly performed in clinical practice, it should also be validated in a spot-time assessment setting to determine the clinical feasibility of its use.

Thus, we investigated whether P-I-R staging has prognostic value in predicting HCC recurrence, a solid clinical endpoint for HCC, in patients undergoing curative resection for CHB-related HCC who had already received at least 2 years of AVT.

## Subjects and Methods

### Patients

Patients who underwent curative resection for CHB-related HCC between 2004 and 2017 at Severance Hospital, Yonsei University College of Medicine, Seoul, Korea, were consecutively enrolled in this study. Surgical resection was performed by four experienced surgeons (DH Han, GH Choi, KS Kim, and JS Choi). Curative resection was defined as satisfaction of all of the following criteria: (1) complete resection of the tumor, (2) negative surgical resection margin according to histopathological examination, and (3) no evidence of residual tumor on computed tomography 1 month after surgery. Of these patients, 145 patients who had received ≥2 years AVT at the time of resection were considered to be eligible.

The exclusion criteria were (1) no fibrosis to significant fibrosis (to enable observation of changes in degree of fibrosis following AVT), (2) co-infection with hepatitis C virus or HIV, (3) alcohol ingestion of ≥40 g/day for >5 years, (4) insufficient clinical data, and (5) viremia not controlled by AVT (HBV DNA >2,000 IU/mL) (Supplementary Fig. 1). CHB was defined as persistence of the serum HBV surface antigen for >6 months and HBV DNA positivity, determined by polymerase chain reaction assay.

The study protocol was consistent with the ethical guidelines of the 1975 Declaration of Helsinki and was approved by the institutional review board of Severance hospital (4-2019-0475). The need for informed consent was waived by the institutional review board because it was a retrospective study.

### Recurrence

The first evaluation of postoperative complications and HCC recurrence was performed at 1 month after surgery. Thereafter, imaging evaluations were performed every 2~3 months^17^. Dynamic computed tomography (CT) was the default imaging modality. However, if there were equivocal nodules or CT was not indicated due to an allergic reaction or renal failure, dynamic magnetic resonance imaging was performed. The levels of tumor markers, including α-fetoprotein or des-γ-carboxy prothrombin, were also determined^18,19^.

The primary endpoint was HCC recurrence after curative resection. Recurrence was diagnosed based on the combined findings of clinical examinations and radiological imaging performed in accordance with the guidelines of the American Association for the Study of Liver Disease^20^ and subclassified as early (<2 years) and late (≥2 years) recurrence^21,22^. We defined overall recurrence to include early and late recurrence. Because early recurrence is known to be associated mostly with tumor factors^23^ and late recurrence is background liver pathology^24^, we selected only patients with late recurrence for further statistical analysis including univariate and multivariate analysis.

### Pathological assessment

**Figure 1:**
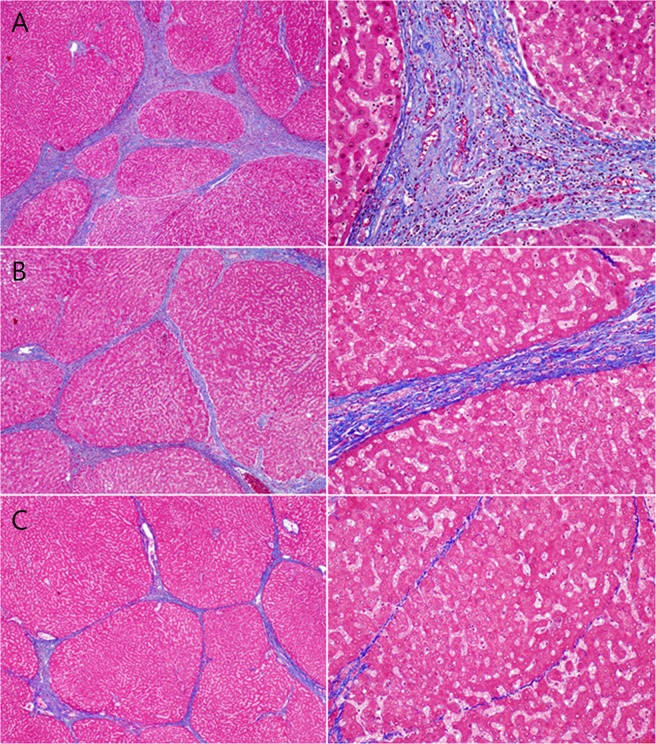
The qualitative fibrosis evaluation using P-I-R staging system was performed. Representative histopathologic features of P-I-R staging showing progressive (**A**), indeterminate (**B**), and regressive (**C**) in patients with chronic hepatitis B taking at least 2 years of antiviral therapy (Masson’s trichrome stain).

The degree of liver fibrosis was evaluated semi-quantitatively according to the Laennec system^6^. Fibrosis was scored as follows: 0, no fibrosis; 1, portal fibrosis; 2, periportal fibrosis; 3, septal fibrosis; and 4, cirrhosis. Cirrhosis was further classified as mild (4A), moderate (4B), or severe (4C) using Laennec staging system^6^. Activity was graded as follows: A0, none; A1, mild; A2, moderate; and A3, severe. The qualitative fibrosis evaluation using P-I-R staging system was performed like followings (Fig. 1). Progressive fibrosis (P) was defined as a case in which >50% of the fibroseptal stroma showed wide/broad loose collagen fibers, a mixture of light and dark trichrome-stained fibers, moderate to marked infiltration of various inflammatory cells and ductular reactions. Regressive fibrosis (R) was defined as a case in which >50% of the fibroseptal stroma shows thin dense collagen fibers, mainly dark trichrome staining and sparse cellularity. Indeterminate fibrosis (I) was defined as an uncertain balance between progressive and regressive fibrosis^15^. According to our study design, fibrosis was assessed once in a single resected liver specimen per patient.

Two experienced pathologists (YN Park and K Na) who were blinded to the patients’ clinical information performed pathological evaluation of resected non-tumoral liver specimens. First, the interpretational reproducibility between the pathologists was assessed. Then, the two pathologists reviewed the slides with discordant P-I-R staging together and reached consensus, which was used for further statistical analysis.

### Noninvasive assessment of liver fibrosis

The aspartate aminotransferase (AST)-to-platelet ratio index (APRI) and fibrosis-4 (FIB-4) score were calculated as additional noninvasive markers for the assessment of fibrosis^25,26^.

### Statistical analysis

Data are expressed as the means ± standard deviations, medians (ranges), and *n* (%), as appropriate. Differences among continuous and categorical variables were examined with Student’s t-test (or the Mann–Whitney test, when appropriate) and the chi-squared test (or Fisher’s exact test, when appropriate). We examined inter- and intra-pathologist agreement rates, which were defined as the percentages of patients for which the same tumor number and treatment response results were obtained. The strength of concordance, based on κ-values, was interpreted as follows: κ < 0.21, poor; κ = 0.21–0.40, fair; κ = 0.41–0.60, moderate; κ = 0.61–0.80, good; and κ > 0.80, excellent.

Univariate and multivariate Cox proportional hazard regression analyses were used to estimate independent predictors of late recurrence. Hazard ratios (HRs) and corresponding 95% confidence intervals (CIs) were calculated. The Kaplan-Meier method was used to examine the cumulative incidence of recurrence based on histological fibrosis stage. All statistical analyses were performed using SPSS version 20.0 software (SPSS Inc., Armonk, NY, USA). A *P-*values < 0.05 were considered to be statistically significant.

## Results

### Baseline characteristics

After excluding 41 patients according to our exclusion criteria, 104 patients who had received ≥2 years AVT at the time of curative resection for CHB-related HCC were selected for analyses. The baseline characteristics of the study population at the time of curative resection are summarized in Table 1. The mean age was 56.3 ± 8.3 years, and males were predominant (74.0%, n = 77). Thirty-two (30.8%) patients had mild steatosis and two (1.9%) showed moderate steatosis. The mean FIB-4 and APRI scores were 3.39 ± 2.76 and 1.47 ± 2.22, respectively. The mean duration of AVT was 62.6 ± 41.7 months. A total of 66 (63.5%) patients received high genetic barrier drug-based AVT (entecavir or tenofovir), and 24 (23.1%) patients had genotypic mutations to lamivudine or adefovir. Hepatitis B e antigen (HBeAg) positivity was noted in 40 (38.5%) patients and the mean HBV DNA level was 1.7 log_10_ IU/mL.

**Table 1:** Baseline characteristics at the time of curative resection of HCC (n = 104).

Variables	Values
**Demographic data**	
Age, years	56.3 ± 8.3
Male gender	77 (74.0)
Body mass index, kg/m^2^	24.1 ± 2.5
Duration of AVT, months	62.6 ± 41.7
High genetic barrier drug-based AVT	66 (63.5)
Genotypic mutation	24 (23.1)
**Laboratory data**	
Platelet count, 10^9^/L	142.7 ± 44.3
Aspartate aminotransferase, IU/mL	62.3 ± 78.5
Alanine aminotransferase, IU/mL	58.9 ± 72.0
Total bilirubin, mg/dL	0.9 ± 0.6
Serum albumin, g/dL	4.1 ± 0.6
Prothrombin time, INR	1.0 ± 0.1
HBeAg positivity	40 (38.5)
HBV DNA, log_10_ IU/mL	1.7 ± 0.9
**HCC stages**	
BCLC A/B/C	101/3/0
pTNM I/II/III/IVA	67/27/7/3
**Fibrosis assessment**	
FIB-4	3.39 ± 2.76
APRI	1.47 ± 2.22

Values are expressed as mean ± SD or n (%).

HCC, hepatocellular carcinoma; AVT, antiviral therapy; INR, international normalized ratio; HBeAg, hepatitis B e antigen; HBV, hepatitis B virus; BCLC, Barcelona Clinic Liver Cancer; pTNM, pathological Tumor, Node, Metastasis; FIB-4, fibrosis-4; APRI, aspartate aminotransferase to platelet ratio index.

At baseline, 27 (26.0%) patients still had a detectable HBV DNA in spite of ≥2 years AVT. Of these, an add-on strategy was applied for three patients according to their resistance profiles; eight patients were switched to a high-barrier drug. The other 16 patients continued previous antiviral drugs. All these patients achieved virological response during the study period.

### Pathological evaluation of fibrosis by P-I-R staging

P-I-R staging was evaluated in cases with at least focal fibrous septa formation or higher stages of fibrous scarring including Laennec stages 3 and 4 fibrosis, and was not feasible in those with less fibrosis (stages 1 and 2). The P-I-R staging of the two pathologists was in agreement in 96 (92.3%) cases, resulting in an excellent agreement rate (κ = 0.867, *P* < 0.001; Supplementary Table 1). After the two pathologists reviewed eight discordant cases together, 11 (10.6%), 46 (44.2%), and 47 (45.2%) patients were finally allocated to the P, I, and R stages, respectively, which was used for the subsequent statistical analysis. P-I-R staging results were compared to quantitative fibrotic burden evaluated by Laennec staging system (Supplementary Table 2) P stage was correlated with Laennec stage 4C, whereas R stage was correlated with Laennec stage 4A (Pearson’s correlation coefficient = 0.895).

### Associations between P-I-R stage and changes in noninvasive surrogates at the time of resection

We evaluated whether the P-I-R stage of non-tumoral liver at the time of HCC resection reflected the changes in noninvasive surrogates (APRI and FIB-4 score), during ≥2 years before resection (Table 2). For this analysis, an increase or decrease in the APRI or FIB-4 score was defined as >10% increase or decrease from the respective baseline.

**Table 2:** P-I-R staging at the time of HCC resection vs. changes in noninvasive surrogate markers during ≥2 years before resection.

Noninvasive surrogates and their changes		P-I-R staging			Agreement rate (%)	κ-value	*P* value
		Progressive	Indeterminate	Regressive			
Surrogates	Changes*	n = 11	n = 46	n = 47			
APRI	Increased (n = 88)	8	40	40	13.5	0.015	0.808
	Maintained (n = 16)	3	6	7			
	Decreased (n = 0)	0	0	0			
FIB-4	Increased (n = 42)	3	21	18	29.8	0.034	0.574
	Maintained (n = 21)	4	8	9			
	Decreased (n = 41)	4	17	20			

*Increased and decreased were defined as >10% increases and decreases from baseline, respectively. Maintained was defined as <10% from baseline.

HCC, hepatocellular carcinoma; APRI, aspartate aminotransferase to platelet ratio index; FIB-4, fibrosis-4.

During the ≥2-year AVT period, the APRI increased in 88 (84.6%) patients and was maintained in 16 (15.4%) patients. In contrast, the FIB-4 score increased in 42 (40.4%) patients, was maintained in 21 (20.2%) patients, and decreased in 41 (39.4%) patients. The agreement rates between P-I-R staging and these noninvasive surrogates were significantly low (13.5–29.8%; Table 2). The P-I-R staging showed poor correlations with changes in the APRI (κ = 0.015, *P* = 0.808) and FIB-4 score (κ = 0.034, *P* = 0.574), respectively.

### Cumulative incidence of late HCC recurrence according to P-I-R staging

**Figure 2:**
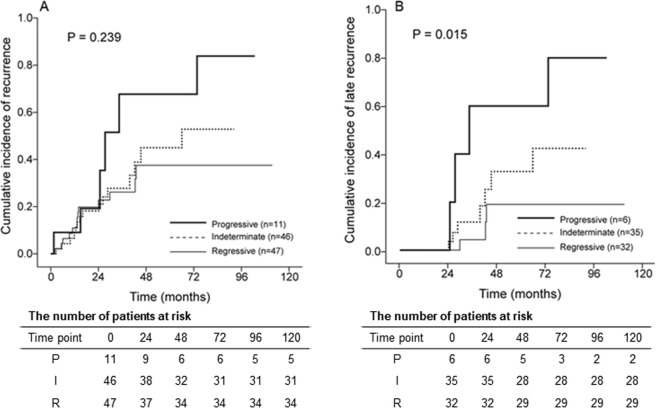
Cumulative incidence rates of HCC recurrence. (**A**) The cumulative incidence of overall recurrence did not differ according to P-I-R staging (*P* = 0.239, log-rank test). (**B**) The cumulative incidence of late recurrence was significantly lower in patients with the regressive pattern than in those with the indeterminate and progressive patterns (*P* = 0.015, log-rank test).

During the follow-up period (mean 45.5 ± 31.7 months), 34 (32.7%) patients experienced HCC recurrence (20 with early recurrence and 14 with late recurrence). Patients with HCC recurrence had similar baseline characteristics, compared to patients without any HCC recurrence except lower total bilirubin (mean 0.8 vs. 1.0 mg/dL, *P* = 0.042) and a higher proportion of HBeAg positivity (31.4% vs. 52.9%, *P* = 0.047) at the time of resection (Supplementary Table 3). The cumulative incidence of overall HCC recurrence according to P-I-R staging was similar (*P* = 0.239, log-rank test; Fig. 2A). Patients with late HCC recurrence had higher alanine aminotransferase (ALT; mean, 59.4 vs. 33.7 IU/mL; *P* = 0.004) and higher HBV DNA level (mean, 2.2 vs. 1.5 log_10_ IU/mL; *P* = 0.002) levels than those without recurrence (Table 3). The cumulative incidence of late HCC recurrence was significantly lower in patients with the regressive fibrosis pattern than in those with the indeterminate and progressive fibrosis patterns of P-I-R staging (*P* = 0.015, log-rank test; Fig. 2B). The progressive group tended to have a higher risk for HCC recurrence than the indeterminate group (*P* = 0.261, log-rank test) and the combined group of patients with indeterminate and regressive fibrosis (*P* = 0.092, log-rank test).

**Table 3:** Comparison of patients with and without late HCC recurrence*.

Variables	No recurrence	Late HCC recurrence	*P* value
	n = 59 (80.8%)	n = 14 (19.2%)	
**Demographic data**			
Age, years	56.8 ± 52.8	52.8 ± 9.4	0.108
Male gender	43 (72.9)	11 (78.6)	0.475
Body mass index, kg/m^2^	23.8 ± 2.5	23.5 ± 2.0	0.671
Duration of AVT, months	69.6 ± 46.4	47.1 ± 20.9	0.082
High genetic barrier drug-based AVT	33 (55.9)	6 (42.9)	0.279
Genotypic mutation	18 (30.5)	5 (35.7)	0.467
**Laboratory data**			
Platelet count, 10^9^/L	144.4 ± 46.4	149.2 ± 46.2	0.730
Aspartate aminotransferase, IU/mL	34.6 ± 23.8	50.4 ± 53.2	0.095
Alanine aminotransferase, IU/mL	33.7 ± 20.9	59.4 ± 51.1	0.004
Total bilirubin, mg/dL	0.9 ± 0.4	0.7 ± 0.3	0.217
Serum albumin, g/dL	4.3 ± 0.5	4.4 ± 0.3	0.344
Prothrombin time, INR	1.0 ± 0.2	1.0 ± 0.1	0.890
HBeAg positivity	22 (37.3)	7 (50.0)	0.298
HBV DNA, log_10_ IU/mL	1.5 ± 0.5	2.2 ± 1.3	0.002
P/I/R stages, n (%)	2 (3.4)/28 (47.5)/29 (49.2)	4 (28.6)/7 (50.0)/3 (21.4)	0.005
**Fibrosis assessment**			
FIB-4	2.74 ± 2.03	2.42 ± 1.93	0.594
APRI	0.81 ± 0.61	1.07 ± 1.30	0.263

Values are expressed as mean ± SD or n (%).

*Patients who died or who were followed-up for less than 2 years were excluded.

HCC, hepatocellular carcinoma; AVT, antiviral therapy; INR, international normalized ratio; HBeAg, hepatitis B e antigen; HBV, hepatitis B virus; FIB-4, fibrosis-4; APRI, aspartate aminotransferase to platelet ratio index.

### Independent predictors of late HCC recurrence

ALT and HBV DNA levels, and regressive fibrosis pattern of P-I-R staging status were significant in the univariate analysis and were selected for the sequential multivariate analysis (Table 4). A higher HBV DNA level was associated independently with an increased risk of late recurrence (HR = 3.200, 95% CI = 1.202–8.518, *P* = 0.020), whereas the regressive fibrosis pattern of P-I-R staging pattern was associated independently with a reduced risk of late recurrence (HR = 0.127, 95% CI = 0.017–0.971, *P* = 0.047).

**Table 4:** Independent predictors of late HCC recurrence*.

Variables	Univariate		Multivariate	
	*P* value	HR (95% CI)	*P* value	HR (95% CI)
Age, years	0.113	0.942 (0.876–1.014)		
Male gender	0.664	1.364 (0.337–5.531)		
Duration of AVT, months	0.099	0.982 (0.960–1.003)		
Platelet count, 10^9^/L	0.725	1.002 (0.990–1.015)		
Alanine aminotransferase, IU/mL	0.018	1.023 (1.004–1.042)	0.131	1.020 (0.994–1.046)
HBeAg positivity	0.411	1.636 (0.506–5.295)		
HBV DNA, log_10_ IU/mL	0.018	2.682 (1.188–6.054)	0.020	3.200 (1.202–8.518)
Alpha fetoprotein, ng/mL	0.942	1.000 (0.994–1.006)		
**Fibrosis assessment**				
FIB-4	0.592	0.904 (0.625–1.307)		
APRI	0.285	1.414 (0.749–2.670)		
F4B, 4 C (vs. F4A, F2-3)	0.057	3.257 (0.965~10.991)		
F4 fibrosis (vs. F2-3)	0.620	1.382 (0.386–4.950)		
**P-I-R staging**				
Regressive (vs. indeterminate + progressive)	0.009	0.088 (0.014–0.544)	0.047	0.127 (0.017–0.971)
Tumor size, cm	0.986	0.993 (0.460–2.143)		
Tumor number	0.911	0.895 (0.128–6.235)		
Edmonson grade 3 (vs. grade 1–2)	0.080	0.292 (0.074–1.158)		
TNM stage II + III (vs. I)	0.253	0.446 (0.112–1.778)		

*A total of 73 (14 patients with late recurrence and 59 patients were followed for more than 2 years without recurrence) were included.

HR, hazard ratio; 95% CI, 95% confidence interval; HCC, hepatocellular carcinoma; AVT, antiviral therapy; HBeAg, hepatitis B e antigen; HBV, hepatitis B virus; FIB-4, fibrosis-4; APRI, aspartate aminotransferase to platelet ratio index; TNM, Tumor-Node-Metastasis.

### Paired pathological data

Paired pathological data were available for 10 patients (Supplementary Table 4). During follow-up, seven (70.0%) patients showed improved in P-I-R staging (from progressive to regressive in three patients and from indeterminate to regressive in four patients). Six (60.0%) patients exhibited regression of fibrosis amount according to the Laennec system (from F4B to F4A in three patients, from F4Cto F4B in two patients, and from F4B to F3 in one patient). No case showed fibrosis progression in P-I-R staging when the patient had received >2 years AVT.

## Discussion

Repeated biopsy to check for dynamic changes in the histological response to AVT is not always feasible in clinical practice. Recently, P-I-R staging, which was developed based on paired liver biopsies was reported to be useful to evaluate the dynamic changes in fibrosis. To improve applicability in clinical practice, one- time assessment of P-I-R staging needs to be validated whether it reflects progressive or regressive fibrosis changes in histological features after prolonged AVT. Thus, we investigated P-I-R staging in non-tumoral liver of CHB in HCC patients with ≥2 years AVT at the time of curative resection. One-time P-I-R assessment predicted the risk of late HCC recurrence (*P* = 0.015), and the regressive fibrosis pattern of P-I-R staging was associated independently with a reduced risk of late HCC recurrence (HR = 0.127).

Several studies have shown the clinical implications of histological subclassification of cirrhosis using the Laennec system, based on the different nodule sizes and fibrous septa thickness^6,19,27^. We confirmed previously that stage 4A is associated with better prognosis than are stage 4B and 4C in terms of the reduced risk of developing decompensation, HCC, or mortality^6^. In contrast to quantitative assessment of fibrosis using the Laennec system, P-I-R staging, qualitative fibrosis evaluation focuses on dynamic changes in the fibrotic burden during prolonged AVT. Interestingly, progressive fibrosis pattern was correlated with Laennec stage 4C, whereas regressive fibrosis pattern was correlated with Laennec stage 4A. In addition, among eight patients showing the regressive pattern at follow-up paired histological assessment, six (60.0%) exhibited regression of fibrosis amount according to the Laennec system. These results suggest that the performance of qualitative fibrosis assessment of P-I-R staging system in addition to quantitative fibrosis evaluation by Laennec staging system would be helpful for comprehensive interpretation of dynamic fibrosis status during AVT (Supplementary Table 2). In our cohort, the presence of steatosis did not affect the risk of HCC recurrence (p = 0.727 by log-rank test). This can be explained in part by that all included patients already had advanced fibrosis, which might offset the effects of the steatosis on HCC recurrence.

Our study has several strengths. First, we used late HCC recurrence as a solid clinical endpoint for longitudinal validation. In contrast, previous studies have involved qualitative assessment of changes in fibrosis at 78 weeks after AVT, but no investigation of the association with prognosis and disease progression in patients with CHB^15,28^. Given the relevance of the association between the fibrotic burden and risk of HCC recurrence^29^, examination of the fitness of any fibrosis stratification through the longitudinal analysis of associations is appropriate. Indeed, our previous study confirmed the fitness of Laennec staging system, which subclassifies histological cirrhosis, revealing differences in the risk of HCC development according to the quantitatively subclassified fibrotic burden^6^. In this study, the qualitative fibrosis evaluation of P-I-R staging was found to predict the late HCC recurrence.

Second, P-I-R staging was conducted with resected specimens in our study. This approach may have almost completely avoided the conventional pitfalls of histological interpretation based on percutaneous liver biopsy and ensured the high reliability of P-I-R staging. In contrast, other studies have been based on P-I-R staging using needle biopsy specimens^15^, which might be vulnerable to a high rate of interpretational variability and sampling error^30^. This issue might explain the higher inter-pathologist reproducibility (κ = 0.867), even with single-time assessment, compared with that reported by Sun *et al*.^15^ for paired biopsy samples (κ = 0.71).

Third, because most patients experience fibrosis regression after appropriate AVT, P-I-R staging, qualitative evaluation of fibrosis is considered to be more clinically relevant in the current era of active AVT compared to classical staging systems with quantitative fibrosis evaluation, which were developed based on histological changes in untreated patients with CHB^16,31^. Our study included a homogenous group of patients with CHB-related HCC, who received ≥2 years AVT at the time of curative resection, but they received a wide spectrum of antiviral agents at the time of HCC resection similar to the actual clinical situation, and not including only entecavir, as in a previous study^28^. The inclusion of low-genetic-barrier drugs might not have influenced our final results, as appropriate rescue therapy for the mutant strain does not affect in the risk of HCC development according to the use of low- or high-genetic-barrier drugs^8^. Furthermore, our follow-up period after curative resection of HCC was long (median 68.1 months), which enabled us to investigate the association between P-I-R staging and the long-term risk of HCC recurrence. Despite exhibiting the regressive fibrosis pattern, three (6.4%, 3/47) patients developed late HCC recurrence. Taken together, these findings indicate that HBV remains still the important cause of HCC development, especially in the presence of advanced fibrosis or cirrhosis^32^.

We are aware of several issues with our study that remains unresolved. First, as a retrospective study, it is not free from potential selection bias. Indeed, only patients with preserved liver function who underwent curative HCC resection were included. In addition, because we evaluated regression and progression of fibrosis using P-I-R staging, the patients with mild fibrosis showing no histologically fibrotic septa cannot be evaluated. Second, based on reliable histological interpretation using resected specimens, we showed high inter-pathologist reproducibility of P-I-R stage even with single assessments. However, the small number of cases (n = 104) who underwent resection after an adequate AVT period of >2 years is a main limitation in our study. The paired biopsy data were taken from an extremely small sample (n = 10), and confirmation of the exact correlation between P-I-R staging and the risk of HCC recurrence was difficult. Third, the mass effect of HCC on the non-neoplastic liver could not be ruled out completely because fibrosis is usually more severe in the liver tissue adjacent to HCC than in background liver tissue far from the mass^33^. As the size and number of HCC nodules that were eligible for curative resection were small, we believe that the tumor effects on fibrosis with respect to surrounding liver histology were likely negligible. In addition, we cannot exclude the possibility that participants who underwent resection due to HCC had greater fibrotic burdens than do actual CHB patients without HCC. Fourth, the cumulative recurrence rate was not different according to Union for International Cancer Control (UICC) stage (*P* = 0.956, log-rank test). This result might be explained in part by that most patients in our cohort had early-stage HCC. Lastly, there was a discrepancy between the changes in APRI and FIB-4 scores. Here, we defined that increased and decreased were defined as >10% increase and decrease from baseline, respectively. Therefore, the changes of these noninvasive fibrosis markers may not be accurately reflected the pathological changes. In addition, weak correlations between P-I-R staining and non-invasive markers might be explained, in part, by APRI and FIB-4 scores’ being unsatisfactory for assessing fibrotic burden according to the Ishak stage^34^.

In conclusion, one-time assessment of P-I-R staging in patients with CHB-related HCC receiving AVT independently predicted the late recurrence of HCC. Therefore, qualitative fibrosis assessment by P-I-R staging in addition to semiquantitative fibrosis evaluation using a previously developed staging system is considered to provide more comprehensive information for the dynamics of fibrosis status and prediction of the outcomes in patients with chronic hepatitis undergoing AVT.

## Supplementary information

Supplementary
