# Supplementary material for: Predictive validation of qualitative fibrosis staging in patients with chronic hepatitis B on antiviral therapy
Source: Sci Rep. 2019 Oct 30;9:15628. doi: 10.1038/s41598-019-51638-3 (PMC6821693; doi:10.1038/s41598-019-51638-3)

**Predictive validation of qualitative fibrosis staging in patients**

**with chronic hepatitis B on antiviral therapy**

Hye Won Lee^1,2,3^, Kiyong Na^4^, Seung Up Kim^1,2,3^, Beom Kyung Kim^1,2,3^, Jun Yong Park^1,2,3^,

Ji Hae Nahm^4^, Jung Il Lee^1,5^, Do Young Kim^1,2,3^, Sang Hoon Ahn^1,2,3^,

Kwang-Hyub Han^1,2,3^, and Young Nyun Park^6^

^1^Department of Internal Medicine, ^2^Institute of Gastroenterology, Yonsei University College of Medicine, Seoul, Republic of Korea, ^3^Yonsei Liver Center, Severance Hospital, Seoul, Korea, ^4^Department of Pathology, Kyung Hee University College of Medicine, Seoul, Korea, ^5^Gangnam Severance Hospital, Yonsei University College of Medicine, Seoul, Korea, ^6^Department of Pathology, Yonsei University College of Medicine, Seoul, Korea.

**Short title**: Validation of qualitative fibrosis staging

Hye Won Lee and Kiyong Na equally contributed to this work.

**Co-corresponding authors**

**Seung Up Kim, MD, PhD**

Department of Internal Medicine, Yonsei University College of Medicine

50-1, Yonsei-ro, Seodaemun-gu, Seoul, 03722, Korea

Tel: +82-2-2228-1944 Fax: +82-2-393-6884 E-mail: [ksukorea@yuhs.ac](mailto:ksukorea@yuhs.ac)

**Young Nyun Park, MD, PhD**

Department of Pathology, Yonsei University College of Medicine

50-1, Yonsei-ro, Seodaemun-gu, Seoul, 03722, Korea

Tel: +82–2–2228–1768; Fax: +82–2–362–0860; E–mail: [young0608@yuhs.ac](mailto:young0608@yuhs.ac)

| **Supplementary Table 1.** P-I-R staging according to fibrotic burden | | | | |
| --- | --- | --- | --- | --- |
|  | Total number of case (n) | Progressive case (n) | Indeterminate case (n) | Regressive case  (n) |
| Stage 1 | 0 | - | - | - |
| Stage 2 | 0 | - | - | - |
| Stage 3 | 37 | 1 | 18 | 18 |
| Stage 4 | 67 | 10 | 28 | 29 |
| 4A | 27 | 0 | 1 | 26 |
| 4B | 31 | 2 | 26 | 3 |
| 4C | 9 | 8 | 1 | 0 |

| **Supplementary Table 2**. P-I-R staging by the two pathologists | | | | |
| --- | --- | --- | --- | --- |
| Pathologist |  | Pathologist B | | |
|  | P-I-R staging | Progressive | Indeterminate | Regressive |
| Pathologist A | Progressive | 6 | 6 | 0 |
|  | Indeterminate | 0 | 43 | 2 |
|  | Regressive | 0 | 0 | 47 |
| Final agreement | | 11 | 46 | 47 |

| **Supplementary Table 3.** Comparison of patients with and without overall recurrence | | | |
| --- | --- | --- | --- |
| Variables | No recurrence | Overall recurrence | *P* value |
|  | n=70 (67.3%) | n=34 (32.7%) |  |
| Demographic data |  |  |  |
| Age, years | 56.8 ± 8.4 | 55.3 ± 8.3 | 0.395 |
| Male gender | 50 (71.4) | 27 (79.4) | 0.267 |
| Body mass index, kg/m^2^ | 24.1 ± 2.7 | 24.1 ± 2.2 | 0.936 |
| Duration of AVT, months | 66.5 ± 42.9 | 54.7 ± 38.5 | 0.179 |
| High genetic barrier drug-based AVT | 47 (67.1) | 19 (55.9) | 0.183 |
| Genotypic mutation | 13 (18.6) | 11 (32.4) | 0.095 |
| Laboratory data |  |  |  |
| Platelet count,10^9^/L | 140.6 ± 46.6 | 147.0 ± 39.5 | 0.492 |
| Aspartate aminotransferase, IU/mL | 67.3 ± 86.6 | 51.9 ± 58.1 | 0.352 |
| Alanine aminotrasferase, IU/mL | 61.4 ± 78.9 | 53.7 ± 56.0 | 0.609 |
| Total bilirubin, mg/dL | 1.1 ± 0.7 | 0.8 ± 0.3 | 0.042 |
| Serum albumin, g/dL | 4.1 ± 0.6 | 4.3 ± 0.4 | 0.051 |
| Prothrombin time, INR | 1.1 ± 0.1 | 1.0 ± 0.1 | 0.136 |
| HBeAg positivity | 22 (31.4) | 18 (52.9) | 0.047 |
| HBV DNA, log_10_ IU/mL | 1.6 ± 0.9 | 1.7 ± 1.0 | 0.588 |
| Fibrosis assessment |  |  |  |
| FIB-4 | 3.69 ± 3.10 | 2.76 ± 1.76 | 0.105 |
| APRI | 1.66 ± 2.57 | 1.08 ± 1.14 | 0.210 |
| Values are expressed as mean ± SD or n (%). | | | |
| AVT, antiviral therapy; HBeAg, hepatitis B e antigen; HBV, hepatitis B virus; FIB-4, fibrosis-4; APRI, aspartate aminotransferase to platelet ratio index. | | | |

| **Supplementary Table 4**. Detailed information from patients with paired histological data (n=10) | | | | | | | | | | | | | | | |
| --- | --- | --- | --- | --- | --- | --- | --- | --- | --- | --- | --- | --- | --- | --- | --- |
| Patient ID | At the time of HCC resection | | | | | | |  | Follow-up histology | | | | | | |
|  | Age | Fibrosis | P-I-R staging | HBV DNA, log10 IU/mL | ALT, IU/mL | FIB-4 | APRI |  | Interval periods from resection (months) | Fibrosis | P-I-R staging | HBV DNA, log10 IU/mL | ALT, IU/mL | FIB-4 | APRI |
| #1 | 50 | 4A | I | 1.3 | 102 | 1.85 | 0.896 |  | 47.9 | 4B | I | 1.3 | 394 | 3.26 | 0.854 |
| #2 | 57 | 4B | I |  | 123 | 2.32 | 0.974 |  | 67.0 | 4A | R | 1.8 | 212 | 3.02 | 0.711 |
| #3 | 49 | 4B | P |  | 41 | 1.77 | 0.680 |  | 81.7 | 4A | R | 1.3 | 123 | 3.32 | 0.771 |
| #4 | 43 | 4C | P | 1.8 | 130 | 2.16 | 0.754 |  | 34.9 | 4B | R |  | 90 | 2.35 | 0.625 |
| #5 | 71 | 4C | P | 1.3 | 67 | 2.63 | 0.598 |  | 51.2 | 4B | R | 1.3 | 29 | 3.46 | 0.553 |
| #6 | 42 | 4B | I | 1.3 | 130 | 1.41 | 0.602 |  | 26.7 | 4A | R | 1.3 | 45 | 1.92 | 0.550 |
| #7 | 54 | 3 | R |  | 74 | 2.74 | 0.730 |  | 30.3 | 4A | R | 1.3 | 17 | 3.83 | 0.982 |
| #8 | 60 | 4B | I |  | 384 | 1.77 | 0.389 |  | 17.3 | 4B | R | 1.4 | 141 | 2.06 | 0.367 |
| #9 | 55 | 4B | I |  | 43 | 3.47 | 1.693 |  | 183.3 | 4B | I | 1.3 | 83 | 1.34 | 0.251 |
| #10 | 45 | 4B | I |  | 127 | 3.49 | 2.164 |  | 50.3 | 3 | R | 1.3 | 90 | 0.98 | 0.332 |
| HCC, hepatocellular carcinoma; FIB-4, fibrosis-4; APRI, aspartate aminotransferase to platelet ratio index. | | | | | | | | | | | | | | | |

**Figure legends**

**Supplementary figure 1**. Flow chart of patient’s enrollment.


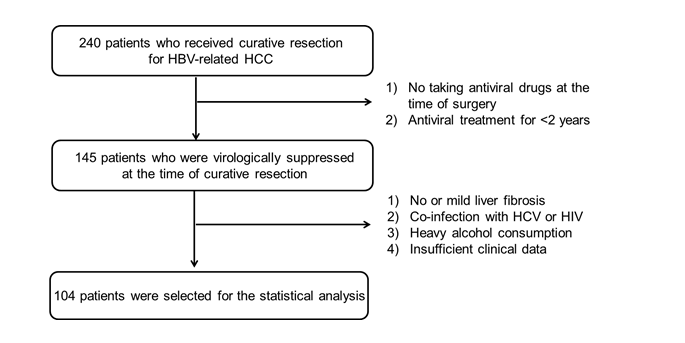

Supplement: Supplementary file 1 — Supplementary [file 41598_2019_51638_MOESM1_ESM.docx]
